# Metabolomic profiling demonstrates evidence for kidney and urine metabolic dysregulation in a piglet model of cardiac surgery-induced acute kidney injury

**DOI:** 10.1152/ajprenal.00039.2022

**Published:** 2022-05-09

**Authors:** Jesse A. Davidson, Justin Robison, Ludmila Khailova, Benjamin S. Frank, James Jaggers, Richard J. Ing, Scott Lawson, John Iguidbashian, Eiman Ali, Amy Treece, Danielle E. Soranno, Suzanne Osorio-Lujan, Jelena Klawitter

**Affiliations:** ^1^Department of Pediatrics, University of Colorado, Aurora, Colorado; ^2^Department of Pediatrics, Washington University, St. Louis, Missouri; ^3^Department of Surgery, University of Colorado, Aurora, Colorado; ^4^Department of Anesthesiology, University of Colorado, Aurora, Colorado; ^5^Heart Institute, Children’s Hospital Colorado, Aurora, Colorado; ^6^Department of Pathology, University of Colorado, Aurora, Colorado

**Keywords:** cardiopulmonary bypass, congenital heart disease, kynurenic acid, metabolism, Warburg effect

## Abstract

Acute kidney injury (AKI) is a common cause of morbidity after congenital heart disease surgery. Progress on diagnosis and therapy remains limited, however, in part due to poor mechanistic understanding and a lack of relevant translational models. Metabolomic approaches could help identify novel mechanisms of injury and potential therapeutic targets. In the present study, we used a piglet model of cardiopulmonary bypass with deep hypothermic circulatory arrest (CPB/DHCA) and targeted metabolic profiling of kidney tissue, urine, and serum to evaluate metabolic changes specific to animals with histological acute kidney injury. CPB/DHCA animals with acute kidney injury were compared with those without acute kidney injury and mechanically ventilated controls. Acute kidney injury occurred in 10 of 20 CPB/DHCA animals 4 h after CPB/DHCA and 0 of 7 control animals. Injured kidneys showed a distinct tissue metabolic profile compared with uninjured kidneys (*R*^2^ = 0.93, *Q*^2^ = 0.53), with evidence of dysregulated tryptophan and purine metabolism. Nine urine metabolites differed significantly in animals with acute kidney injury with a pattern suggestive of increased aerobic glycolysis. Dysregulated metabolites in kidney tissue and urine did not overlap. CPB/DHCA strongly affected the serum metabolic profile, with only one metabolite that differed significantly with acute kidney injury (pyroglutamic acid, a marker of oxidative stress). In conclusion, based on these findings, kidney tryptophan and purine metabolism are candidates for further mechanistic and therapeutic investigation. Urine biomarkers of aerobic glycolysis could help diagnose early acute kidney injury after CPB/DHCA and warrant further evaluation. The serum metabolites measured at this early time point did not strongly differentiate based on acute kidney injury.

**NEW & NOTEWORTHY** This project explored the metabolic underpinnings of postoperative acute kidney injury (AKI) following pediatric cardiac surgery in a translationally relevant large animal model of cardiopulmonary bypass with deep hypothermic circulatory arrest. Here, we present novel evidence for dysregulated tryptophan catabolism and purine catabolism in kidney tissue and increased urinary glycolysis intermediates in animals who developed histological AKI. These pathways represent potential diagnostic and therapeutic targets for postoperative AKI in this high-risk population.

## Graphical abstract

**Figure:**
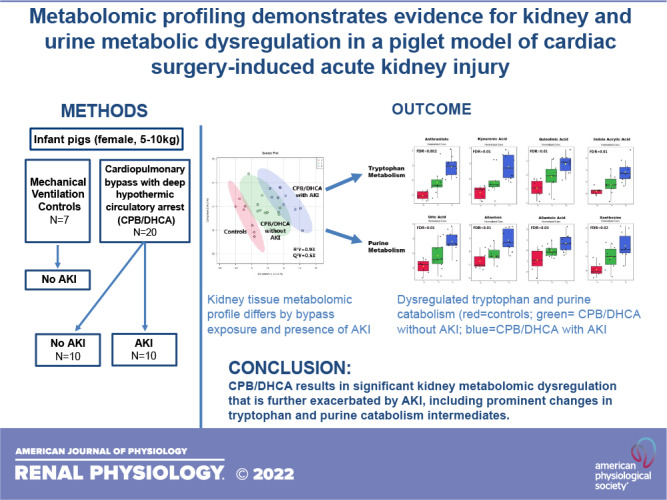

## INTRODUCTION

Acute kidney injury (AKI) is common following congenital heart disease (CHD) surgery with an incidence of >25% (1–5). Postoperative AKI is associated with a sixfold increase in mortality in this population (6–9). AKI also results in postoperative morbidity through multiple mechanisms including fluid overload, endothelial dysfunction, systemic inflammation, and altered clearance of electrolytes, drugs, and metabolites (10–17), leading to increased duration of mechanical ventilation, length of stay, and hospital cost (2, 6, 10). Despite intensive research into the diagnosis and treatment of AKI after CHD surgery, few advances have been made. Clinical diagnosis continues to be based primarily on serum creatinine levels and urine output, whereas treatment remains limited to supportive care, avoidance of nephrotoxins, and, in severe cases, institution of renal replacement therapy. These efforts have been hampered by poor mechanistic understanding and the lack of relevant translational models in which to study this complex process at the organ level (18, 19).

Metabolomics represents a novel approach to the study of AKI (16, 19). Metabolites are highly conserved, low-molecular weight compounds (<1,500 Da) that are the ultimate end products of gene and protein expression. Collectively, these metabolites are called the metabolome and are responsible for cellular energy production and dynamic homeostasis. The kidneys are highly metabolically active with oxygen consumption second only to the heart (20, 21). The primary driver of kidney metabolic activity is tubular transport, and the proximal tubules are at the highest risk for acute postoperative injury (20, 22, 23). However, despite the potential importance of metabolomic dysregulation in the development of postoperative AKI, little is known about the metabolomic changes that occur in injured and uninjured kidneys following cardiac surgery.

Our group and others have begun to characterize the metabolomic changes caused by CHD surgery (24–27). CHD surgery consistently results in profound disruption of both circulating and urinary metabolic profiles (24, 27). We have recently shown that the circulating metabolic profile may also help differentiate between neonates/infants with and without moderate to severe postoperative AKI (28). These metabolic changes included evidence of disrupted purine, cysteine/methionine, and kynurenine/nicotinamide metabolism. Human metabolic profiling studies are limited, however, by the lack of corresponding tissue analysis to confirm histological kidney injury (vs. functional changes in filtration) and determine tissue-level changes in the metabolome.

Animal models are particularly useful for studying metabolomics as metabolic pathways are highly conserved across species, in contrast to protein biomarkers that demonstrate significant heterogeneity. Several studies have sought to identify changes in kidney tissue metabolomic profiles in small and large animal models of ischemia-reperfusion using vascular clamping strategies (29–34). However, concerns have been raised that small animal models do not always accurately reflect human AKI and, in particular, models of kidney ischemia-reperfusion injury may not replicate the complex pathophysiology of human cardiac surgery-associated AKI (18). Translational large animal models of cardiopulmonary bypass (CPB)-associated AKI may help fill this gap (18). We have recently developed a pediatric pig model of CPB with deep hypothermic circulatory arrest (DHCA) in which to study postoperative multiorgan injury, including AKI (35). The model has been shown to induce significant metabolic changes in the lung (36), but the metabolic changes in the kidney and their association with AKI have not been defined. Furthermore, the relationship among tissue, circulating, and urinary metabolic profiles is not known.

In this study, we used our translationally relevant large animal model of pediatric CPB/DHCA in conjunction with targeted metabolic profiling by liquid chromatography-tandem mass spectroscopy (LC-MS/MS) to determine the kidney tissue, urine, and serum metabolic profiles in infant piglets exposed to CPB/DHCA and anesthesia-only controls. Metabolic profiling studies are typically the first stage of metabolomic research (37), used to identify both global changes in the metabolome as well as candidate dysregulated metabolic pathways for further exploration. Our primary goal was to measure kidney tissue metabolic changes and candidate pathways. We hypothesized that kidneys with histological evidence of AKI would demonstrate a tissue metabolic profile that was distinct from both CPB/DHCA-exposed uninjured kidneys and uninjured kidneys from control animals. Furthermore, as secondary goals, we sought to determine whether CPB/DHCA animals with histological AKI could be distinguished from those without AKI by either their early serum or urine metabolic profiles and to what extent the serum and urine metabolic profiles mirrored the metabolic changes seen in the tissue.

## METHODS

### CPB/DHCA Piglet Model

The animals used in this study were part of a parent study on the effects of bovine intestinal alkaline phosphatase infusion on AKI during CPB/DHCA (35). Kidney tissue metabolomic profiling of AKI was a prespecified secondary endpoint of the study. The animal protocol was approved by the Institutional Animal Care and Use Committee of the University of Colorado in accordance with the National Institutes of Health Guide for the Care and Use of Laboratory Animals and Animal Research: Reporting of In Vivo Experiments guidelines. The surgical protocol has been previously published (35). Briefly, we placed infant pigs (5–10 kg, all female pigs) on peripheral CPB through cannulation of the internal carotid artery and external jugular vein. The CPB circuit consisted of a pediatric oxygenator (LivaNova, London, UK), a roller pump, and whole blood prime. Animals were cooled using the CPB circuit to 22°C (rectal), consistently achieving asystole without the need for cardioplegia. CPB was then discontinued, and animals remained in DHCA for 75 min. Following DHCA, CPB was reinitiated, and animals were rewarmed to 36°C. Inotropic support was initiated at 34°C using standardized starting doses of epinephrine, dopamine, and milrinone. Defibrillation was performed as required. Animals were then separated from CPB and provided intensive care unit care for 4 h including mechanical ventilation and inotropic/vasoactive support. An additional group of animals was ventilated under anesthesia for 7 h without undergoing CPB/DHCA to serve as controls. Animals in the parent study were allocated in groups of two to three (three animals per group if a control was included) in rotating order to reduce confounding. Formal randomization was not performed.

### Sample Collection

Immediately before euthanasia, whole blood for serum metabolomic profiling was obtained from the femoral arterial line and urine was obtained by direct bladder puncture. Urine and serum samples were frozen at −80°C for batch analysis. Following euthanasia, the right kidney was harvested, and the tissue was flash-frozen in liquid nitrogen for metabolic profiling or placed in 10% formalin for histology. Serum creatinine, serum and urine neutrophil gelatinase-associated lipocalin (NGAL), and kidney NGAL and kidney injury molecule-1 (KIM-1) mRNA were measured as previously published (35).

### Histology

Kidney tissue fixed overnight in 10% formalin was then paraffin-embedded and sectioned at 4 µm. Sections were oriented to demonstrate a radial section of the cortex and medulla. Serial sections were stained with hematoxylin and eosin and evaluated for the severity of histological kidney injury (proximal tubule injury-primary outcome) by a blinded board-certified pediatric pathologist with a specialization in pediatric kidney disease (A.T.) using a previously published methodology (35, 38–41). Tubular histology was chosen as the primary outcome as it occurs early after injury (before the rise in circulating creatinine) (5, 9, 42) and, unlike filtration measures, its use reduces confounding by physiological changes in renal blood flow (22). At low magnification (×4), sections were evaluated for tubular dilatation and epithelial flattening and scored on a scale of 0–4 based on the percentage of the parenchyma affected, as follows: 0 = none to <5%, 1 = 5–25%, 2 = 26–50%, 3 = 51–75%, and 4 = >75%.

### Sample Processing for Metabolic Profiling

Frozen tissue samples were extracted according to a previously published protocol (36). Frozen samples were ground with a tissue grinder in 500 µL of ice-cold methanol-water [80/20 (vol/vol)], sonicated for 10 min, incubated for 4 h at −80°C for protein precipitation, and centrifuged at 14,000 *g* for 20 min at 4°C. The precipitant was reextracted with an additional 500 µL of ice-cold methanol-water [80/20 (vol/vol)], and the supernatants were combined, centrifuged, and dried in a SpeedVac concentrator (Savant, Thermo Fisher, Waltham, MA). Serum and urine samples (100 µL) were mixed with 400 µL ice-cold methanol, sonicated for 10 min, and incubated for 4 h at −80°C for protein precipitation. This was followed by centrifugation at 14,000 *g* for 20 min at 4°C and drying in a SpeedVac concentrator. All samples were reconstituted with 60 µL of water-methanol [80:20 (vol/vol)] and transferred into HPLC vials with inserts for analysis.

### Mass Spectrometry

Multiple reaction monitoring of 235 metabolites using a positive/negative ion-switching 5500 QTRAP HPLC-MS/MS was used for analysis as previously published (24, 43). The complete list of metabolites and transitions is provided in Supplemental Data S1 (https://doi.org/10.6084/m9.figshare.19666416.v1). Yuan et al. in their study have demonstrated reproducibility and extraction recovery testing using this protocol in formalin-fixed paraffin-embedded normal kidney tissue as well as in kidneys of patients with acute myeloid leukemia. Our samples were immediately frozen and processed quickly using the previously published protocol; thus, no negative impact regarding metabolite stability or recovery was expected.

Sample analysis was performed using an Agilent 1200 series HPLC system (Agilent Technologies, Palo Alto, CA) interfaced with an ABSciex 5500 hybrid triple quadrupole/linear ion trap mass spectrometer (Concord, ON, Canada) equipped with an electrospray ionization source operating in the positive/negative switch mode. The Q1 (precursor ion) and Q3 (fragment ion) transitions, the metabolite names, dwell times, and the appropriate collision energies for both positive and negative ion modes were adapted from a study by Yuan et al. with several metabolite transitions added by our group (validated using pure reference compounds with linearity tested by serial dilution). In total, Q1 and Q3 transitions were set to unit resolution for optimal metabolite ion isolation and selectivity. In addition, the polarity switching (settling) time was set to 50 ms. In 1.42 s using a 3-ms dwell time, we were able to obtain 6–14 scans per metabolite peak. Eight microliters of sample were injected onto an Amide XBridge HPLC column (3.5 μm, 4.6-mm inner diameter × 100-mm length, Waters, Milford, MA). The mobile phases consisted of “HPLC buffer A” [pH 9.0: 95% (vol/vol) water, 5% (vol/vol) acetonitrile, 20 mM ammonium hydroxide, and 20 mM ammonium acetate] and “HPLC buffer B” (100% acetonitrile). The HPLC settings were as follows: from 0 to 3 min, the mobile phase was kept at 85% *solvent B*; and from 3 to 22 min, the percentage of *solvent B* was decreased from 85% to 2% and was kept at 2% for additional 3 min. At *minute 26*, *solvent B* was increased again back to 85% and the column flushed for an additional 7 min at 85% *solvent B*. Once the data were acquired, MultiQuant (v. 2.1.1., Sciex, Foster City, CA) software was used for data processing of 235 unique metabolites. For between-sample normalization, the intensity values for each sample were summed, and the median value of the sums across all samples were determined. Tune and quality control samples were evenly distributed during the batches. The intensity values of each sample were then scaled such that the sum of the scaled intensities equaled the median value of all samples. Urinary metabolites were also normalized to urine creatinine. MS analysis was performed by a blinded investigator (J.K.).

### Statistical Analysis

Sample size was determined by the parent study (dose-finding study for bovine intestinal alkaline phosphatase infusion) (35). All animals from the parent study were used in this prespecified secondary analysis with no exclusions (*n* = 27, 20 CPB/DHCA animals and 7 controls). Our primary outcome was differences in the metabolic profile and individual metabolites of kidney tissue from the following three groups: mechanically ventilated controls, CPB/DHCA animals without AKI (AKI score = 0), and CPB/DHCA animals with AKI (AKI score = 1–4). Secondary outcomes were the difference in the metabolic profile and individual metabolites in serum and urine from the three groups and the comparison of the metabolic profiles of kidney tissue, serum, and urine. Metabolite relative peak intensities (log-transformed and auto-scaled to meet assumptions of normal distribution) were analyzed using MetaboAnalyst 5.0 (http://www.metaboanalyst.ca) (44). Partial least-squares discriminant analysis (PLS-DA) was then used to identify the features that discriminated among the groups of interest. One-way ANOVA was performed to determine which individual metabolites differed in at least one group. The *P* value was adjusted for a false discovery rate of 0.05. When differences were found among groups, Fisher’s least significant difference post hoc test was performed to identify which pairwise comparisons differed significantly. All figures were created using MetaboAnalyst 5.0.

## RESULTS

**Figure 1.**
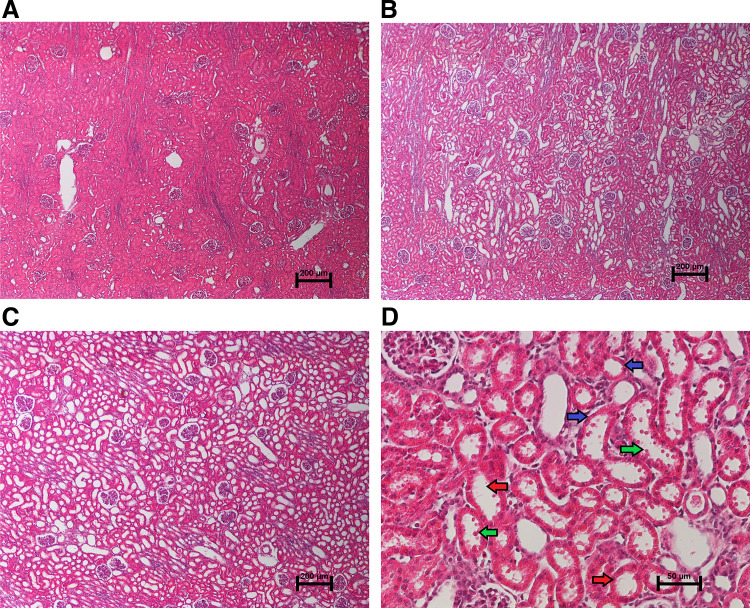
Representative histological findings. Hematoxylin and eosin staining of the renal cortex for acute kidney injury (AKI) histology scoring is shown. *A*: representative section from an anesthesia control animal demonstrating normal histology without evidence of AKI on low magnification (×4). *B*: section from an animal 4 h after cardiopulmonary bypass with deep hypothermic circulatory arrest (CPB/DHCA) demonstrating moderate AKI with 25–50% of the cortical area affected. *C*: section from an animal 4 h after CPB/DHCA demonstrating severe AKI with 75–100% of the cortical area affected. *D*: high-magnification (×20) view of damaged proximal tubules demonstrating proximal tubular dilation (red arrows), epithelial thinning (blue arrows), and epithelial blebs (green arrows). [Adapted with permission from Davidson et al. (35).]

A total of 27 animals were studied (20 CPB/DHCA animals and 7 controls). No control animals developed histological AKI. Of the CPB/DHCA animals, 50% developed histological AKI with proximal tubular injury (loss of the brush border, bleb formation, and tubular dilation) in >5% of tubules on low-powered microscopic examination as previously published (35). Details on the severity of AKI, histology, physiological measurements, and biomarkers of AKI in these animals have also been previously published and are shown here for reference as Table 1 and Fig. 1 (35). By definition, the tissue histology score was higher in the AKI group with a median injury score of 2 (25–50% of the tubules demonstrating histological injury). Serum creatinine did not differ among groups as expected due to the early time point of sampling (4 h post-CPB). CPB/DHCA resulted in significant increases in serum NGAL, the urine NGAL-to-creatinine ratio, and tissue NGAL mRNA as well as a decrease in tissue KIM-1 mRNA, with trends toward further increases in serum NGAL and the urine NGAL-to-creatinine ratio in CPB/DHCA animals with AKI that did not reach statistical significance (35).

**Table 1.** Comparison of tissue histology scores, functional and injury biomarkers, and urine output among mechanical ventilation control animals, CPB/DHCA animals without AKI, and CPB/DHCA animals with AKI

	Controls (*n* = 7)	CPB/DHCA Without AKI (*n* = 10)	CPB/DHCA With AKI (*n* = 10)	*P* Value
Tissue histology score	0	0	2 (2-4)	<0.0001
Serum creatinine, mg/dL	1.1 (0.9–1.5)	1.0 (0.8–1.125)	1.1 (0.875–1.35)	0.5161
Urine output, mL	41.0 (30.0–63.3)	63.5 (45.8–84.0)	45.0 (27.5–67.8)	0.1996
Serum NGAL, ng/mL	112.0 (71.1–125.9)	197.2 (164.3–317.1)	204.4 (181.8–296.9)	0.0098
Urine NGAL-to-creatinine ratio	0.06 (0.02–0.07)	0.58 (0.37–1.15)	0.70 (0.55–0.81)	0.0005
Tissue NGAL relative mRNA	1 (0.93–1.19)	2.25 (1.35–2.76)	1.87 (1.03–6.47)	0.0208
Tissue KIM-1 relative mRNA	1 (0.63–1.35)	0.51 (0.41–1.04)	0.26 (0.21–0.41)	0.0022

All values are presented as medians (interquartile ranges). These results have been previously published in open access format (35) and are reproduced here under the Creative Commons Attribution 4.0 International License. AKI, acute kidney injury; CPB/DHCA, cardiopulmonary bypass with deep hypothermic circulatory arrest; KIM-1, kidney injury molecule-1; NGAL, neutrophil gelatinase-associated lipocalin.

### Comparison of Metabolites Across Sample Types

**Figure 2.**
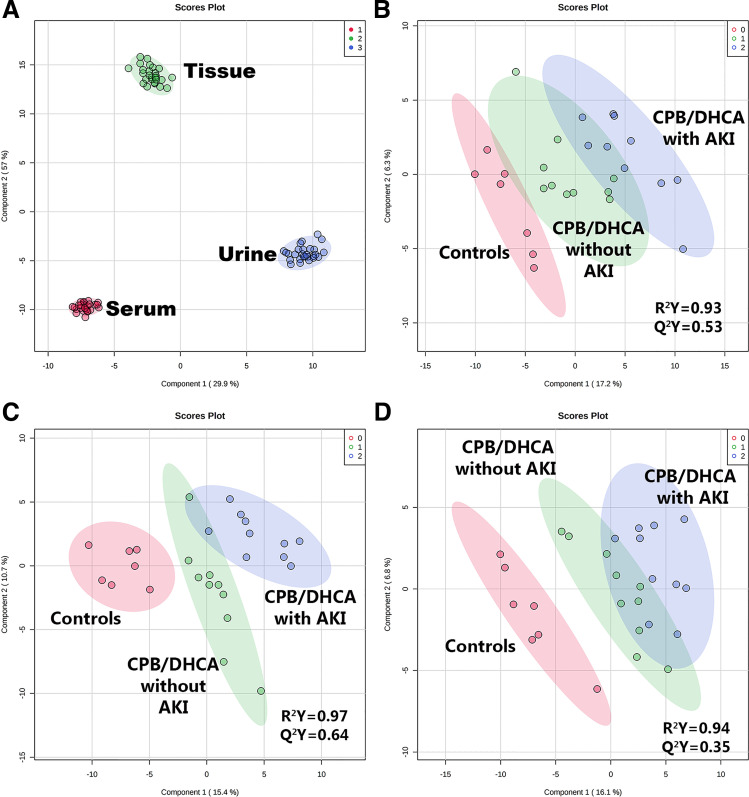
Comparison of metabolic profiles by supervised dimension reduction analysis (PLS-DA). *A*: tissue (green), urine (blue), and serum (red) metabolic profile comparison. PLS-DA demonstrated complete differentiation of tissue, urine, and serum based on their respective metabolic profiles (*R*^2^ = 0.99; *Q*^2^ = 0.99). Comparison of kidney tissue (*B*), urine (*C*), and serum (*D*) metabolic profiles in control animals (*group 0*, red), cardiopulmonary bypass with deep hypothermic circulatory arrest (CPB/DHCA) animals without acute kidney injury (AKI) (*group 1*, green), and CPB/DHCA animals with AKI (*group 2*, blue).

In our metabolomic analysis, we first examined the differences in the tissue, circulating (serum), and urinary metabolic profiles across all 27 animals to assess how closely urinary and circulating metabolites mirrored the relative quantities of metabolites in kidney tissue. Samples clustered very tightly by source (tissue vs. serum vs. urine) on both unsupervised (PCA) and supervised (PLS-DA) dimension reduction analysis (Fig. 2*A*), indicating that each biological medium could be easily distinguished by its metabolic profile (*R*^2^ = 0.99, *Q*^2^ = 0.99). At an individual metabolite level, 98% (231 of 235 metabolites) of the individual metabolites tested differed by sample type at a false discovery rate of <0.05. No difference in the metabolic profile was seen based on exposure to bovine intestinal alkaline phosphatase (*R*^2^ = 0.61, *Q*^2^ = −1.1). The complete metabolite data set is provided in Supplemental Data S2 (https://doi.org/10.6084/m9.figshare.19181945.v1).

### Kidney Tissue Metabolites

**Figure 3.**
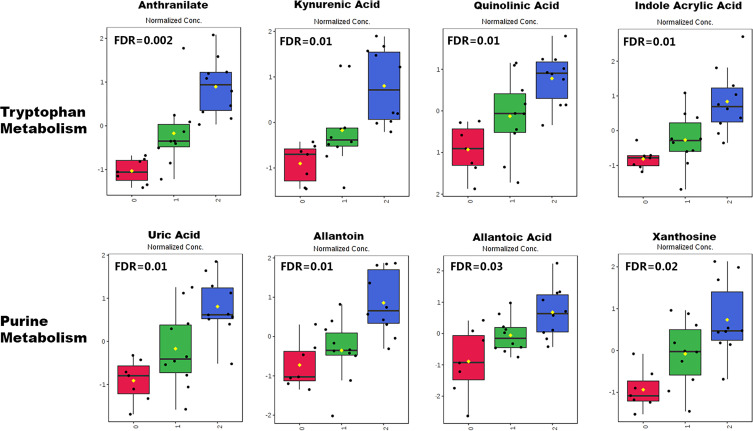
Tryptophan and purine catabolism metabolites in kidney tissue. Multiple tryptophan and purine catabolism metabolites were significantly elevated in cardiopulmonary bypass with deep hypothermic circulatory arrest (CPB/DHCA) animals without acute kidney injury (AKI) (*group 1*, green) compared with controls (*group 0*, red), with further increases seen in CPB/DHCA animals with AKI (*group 2*, blue). FDR, false discovery rate.

Next, we evaluated how the kidney tissue metabolic profile and individual metabolites varied across the following three groups: CPB/DHCA animals with AKI, CPB/DHCA animals without AKI, and mechanically ventilated controls (no AKI). The three groups were readily distinguished by their metabolic profile with moderate reproducibility on cross-validation (*R*^2^ = 0.93, *Q*^2^ = 0.53; Fig. 2*B*). One hundred ninety-seven metabolites measured above the lower limit of detection in kidney tissue (84%). A total of 23 individual metabolites differed across groups by one-way ANOVA with a false discovery rate of <0.05 (Table 2). Of these 23 metabolites, 14 metabolites were significantly elevated on post hoc pairwise testing in kidneys with histological AKI compared with both CPB/DHCA kidneys without AKI and kidneys from controls (Table 2). All 14 metabolites followed the same pattern of change, with the lowest levels in control animals, intermediate levels in CPB/DHCA animals without AKI, and the highest levels in CPB/DHCA animals with AKI. Half of these metabolites arose from two metabolic pathways: tryptophan metabolism (kynurenic acid, anthranilate, quinolinate, and indoleacrylic acid) and purine metabolism (uric acid, allantoin, and xanthosine) (Fig. 3). In addition, even the metabolites that did not reach a statistically significant difference between CPB/DHCA animals with or without AKI still demonstrated the same pattern (Table 2), with the most extreme fold changes seen in CPB/DHCA animals with AKI. Eight of these metabolites were found to have the highest fold changes in CPB/DHCA with AKI animals, whereas a single metabolite (methionine sulfoxide) demonstrated the inverse pattern.

**Table 2.** Kidney tissue, urine, and serum metabolites differing significantly by AKI, CPB/DHCA, or both (FDR < 0.05)

Metabolite	Fold Change CPB/DHCA Without AKI vs. Controls	Fold Change CPB/DHCA With AKI vs. Controls	*P* Value	FDR	Significant Pairwise Comparisons
Kidney tissue					
Significant difference by AKI					
Tryptophan metabolism					
Anthranilate	1.7	2.8	1.4 × 10^−5^	0.002	1-0; 2-0; 2-1
Kynurenic acid	1.6	2.8	4.0 × 10^−4^	0.01	2-0; 2-1
Quinolinate	1.6	2.4	4.7 × 10^−4^	0.01	1-0; 2-0; 2-1
Indoleacrylic acid	1.3	1.9	4.3 × 10^−4^	0.01	2-0; 2-1
Purine metabolism					
Uric acid	2.1	3.9	3.4 × 10^−4^	0.01	2-0; 2-1
Allantoin	1.4	3.7	4.6 × 10^−4^	0.01	2-0; 2-1
Xanthosine	2.0	3.9	8.4 × 10^−4^	0.02	1-0; 2-0; 2-1
Other					
2-Dehydrogluconate	1.4	5.6	2.2 × 10^−5^	0.002	2-0; 2-1
*P*-hydroxybenzoate	1.6	2.7	1.2 × 10^−4^	0.008	1-0; 2-0; 2-1
2-OH-2-methylbutanedioic acid	1.7	6.6	8 × 10^−4^	0.02	2-0; 2-1
4-Pyridoxic acid	1.1	3.3	0.001	0.02	2-0; 2-1
Imidazoleacetic acid	0.87	2.8	0.002	0.03	2-0; 2-1
Pyrophosphate	1.4	2.2	0.003	0.03	2-0; 2-1
Creatinine	1.1	1.4	0.005	0.04	2-0; 2-1
Significant difference by CPB/DHCA exposure only					
3-Methylphenylacetic acid	2.3	3.2	7 × 10^−4^	0.02	1-0; 2-0
Malondialdehyde	1.6	2.1	0.002	0.03	1-0; 2-0
UDP-d-glucose	1.8	2.4	0.003	0.03	1-0; 2-0
Allantoate	1.5	2.4	0.003	0.03	1-0; 2-0
Methionine sulfoxide	0.43	0.34	0.003	0.03	0-1; 0-2
Thiamine	3.6	3.8	0.004	0.04	1-0; 2-0
Hydroxyphenylpyruvate	1.4	2.1	0.004	0.04	2-0
*N*-carbamoylaspartate	1.6	2.5	0.005	0.04	2-0
8-OH-guanosine	2.5	2.7	0.005	0.04	1-0; 2-0
Urine					
Significant difference by AKI					
Ribose-5-phosphate	5.5	9.7	6.7 × 10^−7^	3.1 × 10^−5^	1-0; 2-0; 2-1
AMP	4.1	8.8	8.5 × 10^−7^	3.1 × 10^−5^	1-0; 2-0; 2-1
Putrescine	4.3	10.6	2.2 × 10^−6^	6.9 × 10^−5^	1-0; 2-0; 2-1
α-Ketoglutarate	1.1	1.8	2.0 × 10^−4^	0.003	2-0; 2-1
Phosphoenolpyruvate	1.6	3.2	2.0 × 10^−4^	0.003	1-0; 2-0; 2-1
Phosphorylcholine	1.4	2.7	3.7 × 10^−4^	0.005	1-0; 2-0; 2-1
Glycerophosphocholine	1.4	2.1	5.6 × 10^−4^	0.006	2-0; 2-1
Cytidine	0.76	0.25	0.003	0.02	0-2; 1-2
Glucose-6-phosphate	1.5	3.5	0.004	0.03	2-0; 2-1
Significant difference by CPB/DHCA exposure only					
Lactate	7.6	6.5	6.7 × 10^−9^	1.2 × 10^−6^	1-0; 2-0
Pyruvate	8.2	6.9	1.5 × 10^−8^	1.4 × 10^−6^	1-0; 2-0
Spermine	8.8	12.1	6.1 × 10^−8^	3.7 × 10^−6^	1-0; 2-0
Valine	3.3	4.0	5.9 × 10^−6^	1.6 × 10^−4^	1-0; 2-0
Hypoxanthine	2.3	1.9	1.7 × 10^−5^	4 × 10^−4^	1-0; 2-0
Phenylalanine	2.6	3	6.6 × 10^−5^	0.001	1-0; 2-0
Arginine	3.3	2.8	1.9 × 10^−4^	0.003	1-0; 2-0
Proline	2	1.7	2.3 × 10^−5^	0.003	1-0; 2-0
Glutamine	1.7	2.3	5.2 × 10^−4^	0.006	1-0; 2-0
Citrate	1.8	1.9	8.3 × 10^−4^	0.009	1-0; 2-0
Citrulline	2.7	2.1	9.5 × 10^−4^	0.01	1-0; 2-0
Isocitrate	1.4	1.4	0.001	0.01	1-0; 2-0
Lysine	2.5	2	0.002	0.01	1-0; 2-0
Nicotinate	2.9	2.6	0.002	0.02	1-0; 2-0
Pipecolic acid	1.9	2.3	0.003	0.02	1-0; 2-0
Taurine	2.4	2.5	0.003	0.02	1-0; 2-0
Tyrosine	2.2	2	0.004	0.03	1-0; 2-0
Indole-3-carboxylic acid	1.5	1.8	0.004	0.03	1-0; 2-0
Methionine	1.4	1.7	0.004	0.03	1-0; 2-0
Inosine	1.3	1.2	0.008	0.047	1-0; 2-0
Serine	1.3	1.5	0.008	0.047	1-0; 2-0
Serum					
Significant difference by AKI					
Pyroglutamic acid	0.85	0.73	6.3 × 10^−4^	0.0009	0-1; 0-2; 1-2
Significant difference by CPB/DHCA exposure only					
Inosine	0.09	0.11	8.3 × 10^−8^	1.5 × 10^−5^	0-1; 0-2
Uridine	0.21	0.16	8 × 10^−7^	7.4 × 10^−5^	0-1; 0-2
Adenosine	0.06	0.13	6.1 × 10^−5^	0.003	0-1; 0-2
Methionine	0.55	0.53	7.3 × 10^−5^	0.003	0-1; 0-2
Lactate	1.8	1.9	1.1 × 10^−4^	0.004	1-0; 2-0
Deoxyguanosine	0.32	0.45	1.8 × 10^−4^	0.006	0-1; 0-2
Sedoheptulose-1,7-phosphate	1.9	2.3	3.1 × 10^−4^	0.008	1-0; 2-0
Cytidine	0.51	0.42	3.6 × 10^−4^	0.008	0-1; 0-2
Anthranilate	2.2	4.0	3.9 × 10^−4^	0.008	1-0; 2-0
Malondialdehyde	1.5	1.6	5.5 × 10^−4^	0.009	1-0; 2-0
Arginine	0.66	0.6	6.3 × 10^−4^	0.009	0-1; 0-2
Pyruvate	2.4	2.4	6.4 × 10^−4^	0.009	1-0; 2-0
Glycoxylate	1.7	2.0	6.9 × 10^−4^	0.009	1-0; 2-0
Serine	0.75	0.7	0.001	0.01	0-1; 0-2
AMP	0.01	0.01	0.001	0.01	0-1; 0-2
CTP	0.65	0.56	0.002	0.03	0-1; 0-2
Lipoate	2.8	2.2	0.003	0.03	1-0; 2-0
Malate	1.8	2.1	0.003	0.03	1-0; 2-0

For pairwise comparisons, 0 = mechanically ventilated control group, 1 = cardiopulmonary bypass with deep hypothermic circulatory arrest (CPB/DHCA) animals without acute kidney injury (AKI), and 2 = CPB/DHCA animals with AKI. The first number in each pairwise comparison represents the group with the higher level of the corresponding metabolite (e.g., anthranilate 1-0 = higher level of anthranilate in CPB/DHCA animals without AKI compared with mechanically ventilated controls). FDR, false discovery rate.

### Urine Metabolites

**Figure 4.**
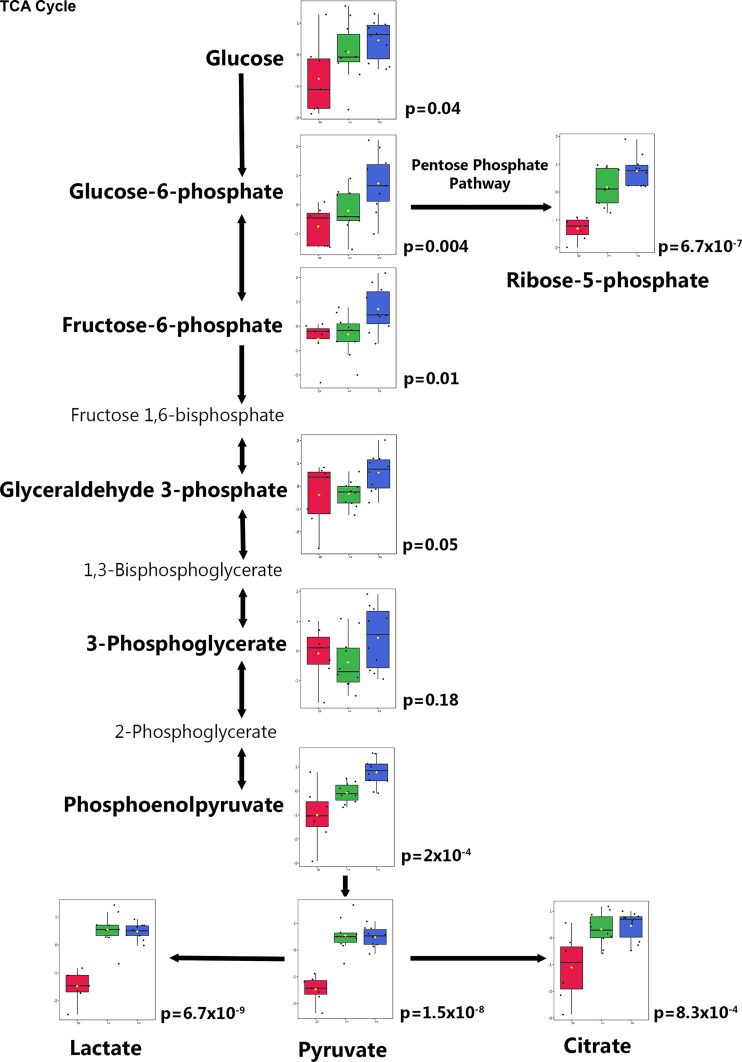
Urine glycolysis intermediates. Glycolysis metabolites from urine in control animals (*group 0*, red), cardiopulmonary bypass with deep hypothermic circulatory arrest (CPB/DHCA) animals without acute kidney injury (AKI) (*group 1*, green), and CPB/DHCA animals with AKI (*group 2*, blue) are shown. Metabolites measured in the metabolic profile assay are shown in bold along with box/whisker plots and *P* values. Intermediates not measured in this metabolic profile assay are shown in Roman (nonbolded) text. Collectively, these data suggest increased glycolytic flux as well as increased entry into the tricarboxylic acid (TCA) cycle, pentose phosphate pathway, and lactate production with CPB/DHCA and potentially further increases with AKI.

The urine metabolic profile also differentiated the three groups of interest (*R*^2^ = 0.97, *Q*^2^ = 0.64), with the most notable separation occurring between mechanical ventilation controls and CPB/DHCA animals (with or without AKI; Fig. 2*C*). One hundred eighty-two metabolites were measured above the lower limit of detection (77%). Of these, 30 metabolites differed among groups by one-way ANOVA (Table 2). Unlike kidney tissue metabolites, most of the significant urine metabolites (21) differed between controls and CPB/DHCA animals rather than based on the presence of AKI. The remaining nine metabolites differed significantly between CPB/DHCA animals with and without AKI. AKI animals demonstrated elevated urine AMP, ribose-5-phosphate, putrescine, glucose-6-phosphate, glycerophosphocholine, phosphoenolpyruvate, phosphorylcholine, and α-ketoglutarate and decreased cytidine (Table 2 and Fig. 4). Intermediates from glycolysis, the tricarboxylic acid (TCA) cycle, and pyruvate metabolism were prominently increased in animals exposed to CPB/DHCA and often further elevated in those that developed AKI, suggesting increased flux through these pathways. Interestingly, we did not identify any overlap in the list of differentially regulated urinary and kidney tissue metabolites. Also, unlike kidney tissue metabolites, urine metabolites that differed significantly only by CPB/DHCA did not show any consistent trends toward differences in the presence or absence of AKI (Table 2).

### Serum Metabolites

The serum metabolic profile provided moderate differentiation among the three groups (*R*^2^ = 0.94, *Q*^2^ = 0.35; Fig. 2*D*). One hundred eighty-five metabolites were measured above the lower limit of detection (79%). Of these, 19 metabolites differed among groups by one-way ANOVA (Table 2). Differences were almost exclusively driven by CPB/DHCA, with all metabolites differing between CPB/DHCA groups (with or without AKI) and controls. Only one metabolite (pyroglutamic acid) was also significantly different between animals with and without AKI. Differences in metabolites associated with CPB/DHCA exposure included increased glycolysis/pyruvate/TCA cycle metabolism intermediates (lactate, pyruvate, glyoxylate, and malate), decreased circulating amino acids (arginine, methionine, and serine), increased markers of oxidative stress (pyroglutamic acid, lipoic acid, and malondialdehyde), and decreased circulating adenosine and AMP. Anthranilate (tryptophan metabolism) was also elevated in CPB/DHCA animals with a trend toward further increases in animals with AKI that did not reach statistical significance.

## DISCUSSION

### Key Findings

This study is the first to examine the concurrent metabolic profiles of kidney tissue, urine, and serum using a complex large animal model of cardiac surgery-induced AKI as supported by the recent National Institute of Diabetes and Digestive and Kidney Diseases working group on overcoming translational barriers in AKI (18). Our novel findings show a distinct tissue metabolic profile of postbypass AKI, including prominent changes in tryptophan and purine metabolism intermediates. Eight urine metabolites also differed significantly in animals with AKI, with a pattern suggestive of increased glycolysis in animals with AKI. Dysregulated metabolites in kidney tissue and urine did not overlap. Serum metabolites measured at this early time point were predominantly affected by CPB/DHCA, and only a single serum metabolite differed based on AKI.

### Kidney Tissue Metabolites

The most prominent findings at the tissue level included increased tryptophan and purine metabolism intermediates. Levels of these metabolites were consistently highest in the kidneys with histological AKI, whereas kidneys from animals exposed to CPB/DHCA but without histological AKI demonstrated intermediate elevations compared with controls. Higher metabolite levels could occur in injured kidneys either due to a synergistic effect of AKI and CPB/DHCA on kidney metabolism or because higher metabolite levels contribute directly to AKI. It is also possible that subtle kidney injury was present in some CPB/DHCA animals that did not manifest on histology but was sufficient to alter tryptophan and purine metabolism.

The kynurenine pathway (KP) is the primary route of tryptophan catabolism. It produces multiple biologically active intermediates, and its end product, quinolate, is the substrate for de novo nicotinamide adenine dinucleotide (NAD) production. During pathological inflammatory states, flux through the KP is increased due to the upregulation of the first pathway enzyme (indolamine 2,3-dioxygenase) across multiple organs (45–48). Evidence points to a potential role for the KP in noncardiac surgery-associated AKI (49). Observational studies in adults with AKI from a mix of etiologies have identified increased circulating kynurenic acid and kynurenine/tryptophan ratios in subjects with AKI (50–54). Several studies also found increases in downstream KP metabolites with AKI (52, 54). At the tissue level, both ischemia-reperfusion injury (vascular clamping) (29, 30) and sepsis (55, 56) lead to increased kidney tissue and circulating KP metabolites in mice. Downregulation of distal KP enzymes also occurs following kidney ischemia-reperfusion, leading to an accumulation of the downstream metabolite quinolate and decreased NAD production (57).

It is less clear if this increase in KP metabolites represents a pathological or counterregulatory process (or a mix of both) in noncardiac surgery-associated AKI. Administration of exogenous kynurenic acid decreases ischemia-reperfusion-induced AKI in mice (58), and blockade of the KP at kynurenine 3-monooxygenase with shunting toward kynurenic acid has a similar effect (29). These findings suggest that kynurenic acid may be protective in isolated ischemia-reperfusion injury, likely through a combination of antioxidant, anti-inflammatory, and *N*-methyl-d-aspartate receptor antagonist properties (49). Reduction of downstream oxidant, proapoptotic, *N*-methyl-d-aspartate receptor agonist KP metabolites may also be beneficial. Blockade of α-amino-β-carboxymuconate-ε-semialdehyde-decarboxylase (the enzyme leading toward picolinic acid production and away from quinolate) also reduces AKI in both ischemia-reperfusion and cisplatin models via an increase in de novo NAD production (59), whereas decreased quinolinate phosphoribosyltransferase increases quinolate, decreases NAD, and results in worsening AKI (57).

For the first time, we now have evidence in both pediatric patients and our complex large animal model of infant CPB/DHCA that the KP is also activated in cardiac surgery-induced AKI. Infant cardiac surgery results in a significant increase in proximal KP metabolites that peaks shortly after surgery and returns to baseline in most cases by 48 h postoperatively (25). Circulating kynurenic acid levels are higher at 24 h postoperatively in patients who subsequently develop moderate-to-severe AKI (28). In the present study, we found that activation of the KP is prominent in kidney tissue, with smaller changes in serum KP metabolites and no significant change in urinary KP metabolites, suggesting primary kidney production rather than filtration of systemically produced KP metabolites. Furthermore, we found that the most distal metabolite in the KP, quinolate, was also highest in kidneys with histological AKI, indicating increased flux through the entire KP. This study did not, however, directly evaluate the causal link between KP activation and AKI after CPB/DHCA. Additional studies are needed to perform a comprehensive quantitative mapping of the KP in this model and assess the effect of pathway modulation on the occurrence and severity of AKI.

Our second prominent finding in injured kidney tissue was an increase in the end products of purine catabolism, particularly xanthosine, uric acid, and allantoin, which has not previously been identified in cardiac surgery-induced AKI. Uric acid is produced primarily in the liver and excreted through the kidney after sequential reabsorption and secretion in the proximal tubule (60). Uric acid is also a major systemic antioxidant in the extracellular environment, with allantoin as the end product (61). In pigs and other nonprimate mammals, allantoin can also be produced enzymatically from uric acid via uricase (uricase is nonfunctional in humans) (60). Serum uric acid has been well described as both a marker of AKI and a contributor to AKI, either through crystal formation or crystal-independent mechanisms (60, 61). Less is known about kidney tissue purine metabolism during AKI. Using untargeted metabolomics, Wei et al. (30) in their study demonstrated increased uric acid and allantoin in the renal cortex of mice that had undergone bilateral kidney vascular clamping compared with sham controls. This increase began early after reperfusion (within 2 h) and was not accompanied by a significant change in plasma uric acid or allantoin. These findings could be due to decreased kidney excretion. Alternatively, uric acid accumulation could directly contribute to AKI through crystal-independent prooxidant and proinflammatory mechanisms (61). Interestingly, in our prior study of children undergoing cardiac surgery with CPB, low circulating uric acid and high allantoate levels at 24 h postoperatively were associated with subsequent AKI (28), and we found a similar nonsignificant trend of decreased serum uric acid and increased allantoin and allantoate in our pigs with AKI (data not shown). Collectively, these findings suggest that although both circulating and tissue purine metabolites may be related to cardiac surgery-induced AKI, the mechanisms may be distinct (e.g., systemic depletion of circulating uric acid through oxidation to allantoin and allantoate vs. local accumulation and potentially toxic effects in kidney tissue). Additional studies are warranted to elucidate both the mechanism of purine metabolite dysregulation and the potential direct effects on the development of AKI after cardiac surgery.

### Urine Metabolites

Unlike our findings in kidney tissue, the urinary metabolites that most strongly differed by exposure to CPB/DHCA and AKI were concentrated within glycolysis and the proximal TCA cycle. These metabolites were typically elevated in animals exposed to CPB/DHCA with further elevation in animals demonstrating histological AKI. Both lactate and ribose-5-phosphate (pentose phosphate pathway) were also increased despite adequate oxygen delivery postbypass and no evidence of impaired flow into the TCA cycle, consistent with increased glycolytic flux and the Warburg effect (aerobic glycolysis). To our knowledge, the Warburg effect has not previously been identified in cardiac surgery-induced AKI. The Warburg effect has been well described in oncology, however, and there is increasing evidence that it may also occur in less complex small animal models of noncardiac surgery-induced AKI. Septic and ischemia-reperfusion models of AKI result in the upregulation of glycolysis and decreased gluconeogenesis (21, 62). Furthermore, inhibition of glycolysis with 2-deoxyglucose improves mitochondrial function (63) and reduces AKI (64) in septic mice.

It is not clear from our study why glycolysis intermediates were significantly altered in urine but not in the kidney tissue itself. This discrepancy could be related to differences between intracellular metabolite concentrations and the effects of altered proximal tubular function on urinary metabolite concentrations. In addition, changes in tissue metabolites could reflect more severely injured, nonfunctional nephrons that do not contribute substantially to urine production. Even though changes in urine metabolites do not directly reflect tissue metabolite changes in this model, our findings suggest that urine biomarkers of increased glycolysis could be promising candidates for early AKI diagnosis after pediatric cardiac surgery. Furthermore, modulation of glycolysis should be further explored in translational models of cardiac surgery for the prevention of AKI.

### Serum Metabolites

Serum metabolites were the least associated with AKI in this study, with only a single metabolite (pyroglutamic acid, a marker of oxidative stress) that was significantly different in animals with histological AKI. Instead, differences in serum metabolic profiles across groups could largely be attributed to exposure to CPB/DHCA at this early time point. This finding is not surprising. We have previously demonstrated that infant cardiac surgery with CPB results in a profound shift in the circulating metabolome that continues to diverge from baseline for at least 24 h (24). This metabolic shift is homogenizing, with all patients showing a similar initial metabolic response to CPB. Thus, it may take time for metabolic changes originating from organ injury to manifest in circulation. Also, the circulating metabolome reflects contributions from multiple metabolically active organs, making early differentiation of single organ injury challenging. In our prior study, infants who subsequently developed KDIGO stage 2/3 AKI could be differentiated based on their 24-h circulating metabolomic profile (28). In our present model, injured kidneys demonstrate clear metabolic derangements as early as 4 h after separation from bypass. Longitudinal studies are needed in this model to help determine whether serum sampled at later time points (4–24 h postoperatively) will better help identify AKI due to either progression of the underlying kidney injury or unmasking of the metabolic effects of AKI as other organs return to preoperative baseline.

### Translational Modeling of AKI Metabolomics After Cardiac Surgery

Collectively, our findings highlight the opportunity created by using a large animal translational model to study kidney metabolomics and AKI after cardiac surgery. Most of our current understanding of kidney metabolic changes in AKI comes from either nonbypass small animal models (sepsis or renal vascular clamping) (29, 30, 65, 66) or human studies of circulating and urine metabolites in critically ill patients (26, 50, 52, 62, 67). As previously discussed, the recent report from the National Institute of Diabetes and Digestive and Kidney Diseases on overcoming translational barriers in AKI noted that existing ischemia-reperfusion injury models are poor representations of cardiac surgery-induced AKI and advocated for use of more translationally relevant models (18). Our model recreates much of the complexity of pediatric cardiac surgery while allowing concurrent measurement of kidney tissue, circulating, and urinary metabolites as well as histological proximal tubular injury. Interestingly, even among large animal models, metabolomic responses may be disease specific, as a recent study of metabolic changes in a porcine model of sepsis-induced AKI did not identify significant dysregulation of either KP or purine catabolism intermediates (68). Similar to this porcine model of sepsis-induced AKI, our findings show that although urine and serum metabolites could help identify animals with AKI, they are not exact mirrors of metabolism at the tissue level. Thus, models capable of measuring all three compartments simultaneously in a complex physiological environment are needed, both for the exploration of metabolic mechanisms of AKI and for the development of metabolic biomarkers/therapies.

### Limitations

Our study has several important limitations. First, the sample size is small compared with human metabolomic studies, limiting the power to detect true differences in metabolites with smaller fold changes and/or greater variability. The consistent finding of greater fold changes in metabolites from injured kidneys even when not statistically different suggests the presence of type 2 error. Second, kidney injury in this model is heterogeneous, and sampling variability itself could lead to misclassification. Future studies should be scaled to both increase the number of animals assessed and to allow for the comparison of multiple samples from both kidneys. Also, cell-specific metabolism changes should be defined using microdissection techniques. Third, this study focused on early kidney injury (4 h post-CPB) to understand the initial metabolic changes at a point where intervention might be more impactful. We did not define the natural history of metabolic changes following CPB/DHCA. Longitudinal studies with repeat urine/serum sampling, continuous functional measurements of filtration, and different euthanasia time points are necessary to understand the progression of metabolic changes, including through the recovery period. Finally, to decrease variability, only female animals were used in this study. We recently demonstrated sex differences in metabolomic and cardiorenal outcomes in a murine model of ischemic AKI (69). Future studies should include both male and female animals, with appropriate power to identify the importance of sex as a biological variable in the metabolic response to CPB/DHCA.

### Perspectives and Significance

With our persistent failure to decipher postoperative organ injury after cardiac surgery, the scientific community is increasingly realizing that complex pathophysiology may require a matching complex research strategy to drive progress (18). In this study, we merged a large animal model of pediatric cardiac surgery with an unbiased molecular big data/omics approach and applied it to the study of postcardiac surgery AKI. Using this strategy, we identified three key dysregulated metabolic pathways (tryptophan catabolism, purine catabolism, and glycolysis) that differentiate animals with postoperative AKI from those who retain kidney health despite the global metabolic stress of CPB/DHCA and may prove to be either drivers of AKI or protective responses. Satisfyingly, these pathways are also dysregulated in simple small animal models and in critically ill humans, suggesting a true biological signal rather than false-positive findings. Translation from bench to bedside should now be taken in a stepwise approach. Key next steps include a systematic shift from relative to absolute metabolite quantification and the development of assays to identify and measure all metabolites within these pathways in a validated manner. Adjacent pathways, such as the nicotinamide pathway downstream from tryptophan catabolism, must similarly be evaluated. Once the pathways have been completely mapped, then experimental pathway modulation can evaluate the role of individual pathway metabolites and pathway flux on end-organ injury, identifying targeted therapies for phase 1 clinical trials.

## SUPPLEMENTAL DATA

10.6084/m9.figshare.19666416.v1 Supplemental Data S1: https://doi.org/10.6084/m9.figshare.19666416.v1;

10.6084/m9.figshare.19181945.v1 Supplemental Data S2: https://doi.org/10.6084/m9.figshare.19181945.v1.

## GRANTS

This work was supported by the Department of Defense Award PR152240 [to Principal Investigator (PI): J.A.D], American Heart Association Grant 17IRG33410724 (to PI: J.A.D.), and National Heart, Lung, and Blood Institute Grants K23HL123634 (to PI: J.A.D.) and R01HL156936 (to PI: J.A.D.).

## DISCLOSURES

No conflicts of interest, financial or otherwise, are declared by the authors.

## AUTHOR CONTRIBUTIONS

J.A.D., J.J., S.L., D.E.S., S.O.-L., and J.K. conceived and designed research; J.A.D., J.R., L.K., S.O.-L., and J.K. performed experiments; J.A.D., L.K., A.T., and J.K. analyzed data; J.A.D., L.K., A.T., S.O.-L., and J.K. interpreted results of experiments; J.A.D. prepared figures; J.A.D. drafted manuscript; J.A.D., J.R., L.K., B.S.F., J.J., R.J.I., S.L., J.I., E.A., A.T., D.E.S., S.O.-L., and J.K. edited and revised manuscript; J.A.D., J.R., L.K., B.S.F., J.J., R.J.I., S.L., J.I., E.A., A.T., D.E.S., S.O.-L., and J.K. approved final version of manuscript. 
